# The Poor Law Infirmary

**Published:** 1911-10-14

**Authors:** 


### THE POOR LAW INFIRMARY.

(Experiences and Suggestions will be welcomed.)

### What it is and What it might be.

In one sense, the best types of these institutions prove that neither money nor brains have been spared so far as the buildings and much of the equipment are concerned. Many a ward in a Poor Law infirmary, structurally considered, apart from its decorative features, is well up to the modern standard. It labours under the great disadvantage of having to accommodate every type to be met with in the lower and poorer grades of the population. The moral difficulties and objections which formerly attached to these infirmaries have in the case of the best administered of them been overcome by a careful classification of the patients. In such well-administered infirmaries the risks and discomforts which once affected the younger and more innocent of the patients have been reduced to a minimum by classification. In those infirmaries where strict and carefully organised classification is not at present found the evils attending the mingling of the innocent and moral with the immoral and degraded still prevails, to the shame of those who permit this unjust and disgraceful state of affairs to continue.

Formerly, although attempts had been made to improve the quality of the nursing by the appointment of a lady superintendent or a superintendent nurse, the system often proved in practice anything but a success, because these skilled officials were under the control of the master or mistress of the workhouse, or both. It would not be difficult to recall instances of grave mishaps and faulty administration due to such a system, which was opposed to reason and justice and did but mark the incapacity of the Boards of Guardians which permitted such a system to continue. Thanks in great measure to the influence of the Local Government Board the position of the lady superintendent has been so improved as to enable her to organise her department and make it efficient within certain limitations, to the great advantage and comfort of the patients and the raising of the standards of efficiency of the nursing and domestic departments of those Poor Law infirmaries which possess such a system. It is not too much to say, however, that many a Poor Law infirmary would be greatly improved, and its status as a hospital immeasurably heightened, if the position and status of the lady superintendent and of her assistants were to be raised everywhere to the standard which now prevails in the best administered of the voluntary hospitals.

Mr. John Burns has rendered good service to the nation and to the poor by the reforms which he has promoted in connection with the improved attendance and care of the sick in many Poor Law institutions. The appointment of a number of carefully selected, educated, and trained women as Poor Law inspectors was a promising conception, but in practice it is not doing the good which everybody hoped would result from it—for the reason, that, the position of these women inspectors, intentionally or for some other reason, is not really independent in practice. We gather, from inquiries we have made, that some of the women inspectors, at any rate, look to and take their instructions as to how to perform their duties, how to act in certain circumstances, and the points which their reports should cover, to the male inspectors in the districts to which they have been attached. We hold, as every hospital administrator holds, that the first duty of the women inspectors should be to win the confidence of the ladies-superintendent and superintendent nurses engaged in Poor Law work and to make themselves familiar with the difficulties with which the heads of the nursing departments are at present confronted, and the causes which have created these difficulties. Then, after due consideration, with a full knowledge of the facts, they should report upon them and recommend such changes and reforms as would not only remove existing difficulties and friction, but secure the maximum of efficiency throughout the Poor Law nursing service. Such a practice would soon give to the heads of that service the independence and position without which it can never be made to equal the efficiency of the sister services in voluntary, naval, and military hospitals.

We have not space to-day to deal with the relations which ought to exist, sometimes do, but, alas! very frequently do not exist in Poor Law infirmaries and kindred establishments. Our regret that we must delay a consideration of these matters and the causes which underlie them is tempered by the hope, that some of the more experienced and able of the medical superintendents, matrons, and ladies-superintendents will spontaneously offer to co-operate with us in fully dealing with all these points with the object of formulating a simpler and better plan than that of many now in force, so that the efficiency of the Poor Law infirmary may be made more nearly to approximate that of the best voluntary hospitals. There are a number of medical superintendents who do appreciate warmly the advantages attaching to a well-organised and ably administered nursing department. There are a number of the ablest and best administrators which the nursing profession contains to be found amongst the ladies-superintendent of Poor Law infirmaries. Nurses who have obtained their training in this service are amongst the most competent of private nurses to be met with in this country. The patients in the wards of Poor Law infirmaries being who they are, are entitled to the maximum of efficiency in treatment and service which knowledge and training can provide. For all these reasons and many others we invite the co-operation of every experienced worker in the Poor Law service, that the maximum of efficiency, with the maximum of comfort too, may speedily find a lasting home within the walls of every Poor Law infirmary throughout the United Kingdom.

